# Test–Retest Reliability of Ankle Mobility, Balance, and Jump Tests in Amateur Trail Running Athletes

**DOI:** 10.3390/sports13100352

**Published:** 2025-10-04

**Authors:** Alberto Dominguez-Muñoz, José Carmelo Adsuar, Santos Villafaina, Juan Luis Leon-Llamas, Francisco Javier Dominguez-Muñoz

**Affiliations:** 1 Facultad de Ciencias del Deporte, Universidad de Extremadura, 10003 Cáceres, Spain; alberto.dm_23@hotmail.com; 2 BioErgon Research Group, Didáctica de la Expresión Musical, Plástica y Corporal, Facultad de Ciencias del Deporte, Universidad de Extremadura, 10003 Cáceres, Spain; jadssal@unex.es; 3 Instituto de Investigación e Innovación en Deporte (INIDE), Universidad de Extremadura, 10003 Cáceres, Spain; svillafaina@unex.es; 4 Grupo de Investigación en Actividad Física, Calidad de Vida y Salud (AFYCAV), Didáctica de la Expresión Musical, Plástica y Corporal, Facultad de Ciencias del Deporte, Universidad de Extremadura, 10003 Cáceres, Spain

**Keywords:** reliability, test–retest, functional tests, trail running

## Abstract

This study aimed to test the reliability of seven functional performance tests in amateur trail runners, including ankle mobility, balance, hopping, and countermovement jump (CMJ) tests. The sample consisted of 35 runners who were evaluated in two sessions separated by 7 to 14 days, which varied due to participants’ scheduling constraints. Relative reliability was assessed using the Intraclass Correlation Coefficient (ICC, which indicates consistency between repeated measures), the Standard Error of Measurement (SEM, which reflects measurement precision), and the Minimal Detectable Change (MDC, which represents the smallest real change beyond measurement error). The results show high reliability in almost all tests. The Lunge Test obtained an ICC of 0.990 and 0.983 for distance, and 0.941 and 0.958 for angular measurements in both legs. The Hop Tests showed moderate reliability with ICC above 0.7 In contrast, the Y Balance Test demonstrated lower reliability, with ICC values ranging from 0.554 to 0.732. The CMJ test showed good reliability, with an ICC ranging from 0.753 to 0.894, an SEM between 5.79% and 11.3%, and an MDC ranging from 15.54% to 31.44%, making it useful for assessing lower limb explosive strength. Both tests presented comparatively higher error values, which should be considered when interpreting individual changes. These findings support the use of these tests as valid and reliable tools for evaluating ankle dorsiflexion, balance, functional symmetry, and lower limb explosive strength in amateur trail runners, prior to training programs or injury prevention strategies, provided that standardized protocols and validated measuring instruments are used.

## 1. Introduction

Trail running is an athletic discipline consisting of running on trails in natural environments, where athletes face uneven terrain, steep gradients, and changing environmental conditions [1]. This sport requires specific physiological and biomechanical adaptations [2,3] and a high level of physical and mental demand, which has drawn the attention of both the scientific community and the field of sports performance.

The factors that determine performance in trail running are similar to those in flat running. According to di Prampero’s classical model for long-distance races, performance depends on VO_2_max, the Maximal Aerobic Speed (MAS), the fraction of this capacity that can be sustained during competition and running economy [4]. In trail running, two additional factors come into play: muscle strength, particularly of the knee extensors [5], and biomechanical adaptability to the terrain [6].

Cardiorespiratory indicators such as VO_2_max, the MAS, running economy, and the anaerobic threshold are well-established predictors of endurance performance in trail and mountain running [5,6,7,8,9,10,11,12]. However, although some of these parameters can be measured with relative ease, their assessment usually requires laboratory equipment and specialized personnel, making them generally more costly and less practical for routine monitoring. This limitation has increased the interest in functional field tests, which can provide complementary and more accessible information on performance and injury risk in trail runners.

Muscle strength, particularly in the knee and hip extensors, is essential in trail running to overcome terrain irregularities. Heavy-load strength training has been shown to improve performance in runners and cyclists [13], increase fatigue resistance [5], and enhance running economy in endurance athletes [14]. Likewise, biomechanical adaptability to terrain enables athletes to respond effectively to environmental variability, promoting efficiency, optimizing effort management, and reducing injury risk [3,15].

Several studies indicate that ankle dorsiflexion predicts running economy and injury prevention [16]. Similarly, both static and dynamic balance, stability, and postural control are critical when navigating technical terrains [17]. Core, hip, knee, and ankle stability are key to force transmission and injury prevention. Poor core stability can alter knee biomechanics [18], while reduced hip stability may result from muscular weakness or tightness [6]. In addition, lower-limb explosive strength is fundamental for overcoming obstacles and steep inclines encountered in natural environments [19].

Given the importance of these factors, valid and reliable assessment tools are necessary. Four field tests with demonstrated reliability in other contexts are particularly relevant for trail running: the Weight-Bearing Lunge Test (WBLT), the Y Balance Test (YBT), the Hop Tests, and the Countermovement Jump (CMJ). Rather than focusing only on performance outcomes, these tests allow the evaluation of ankle mobility, dynamic balance, functional symmetry, and explosive strength—key abilities for negotiating uneven terrain, reducing injury risk, and monitoring training adaptations in trail runners [20,21,22,23,24]. Although these tests have proven reliable in other fields, it is necessary to confirm their validity in trail running due to the sport’s specific demands.

Validating these tools requires verifying their reliability through the test–retest procedure, which consists of performing the same test on two separate occasions and comparing the results. This method allows for the detection of measurement consistency [25], differentiation between application errors and true variability [26], and calculation of the Intraclass Correlation Coefficient (ICC), which is essential for evaluating relative reliability [27].

The aim of the present study is to determine the test–retest reliability of the WBLT, YBT, Hop Test, and CMJ in amateur trail runners. We hypothesize that these tests would show good-to excellent absolute and relative reliability in this population.

## 2. Methodology

### 2.1. Sample Size Calculation

A sample size of 12 participants with 2 observations per participant achieves a statistical power of 91% to detect an ICC of 0.9 (excellent) under the alternative hypothesis, when the intraclass correlation under the null hypothesis is 0.5 (moderate) [28], using an F-test with a significance level of 0.05. The G*Power software (version 3.1.9.7; Heinrich-Heine-Universität Düsseldorf, Düsseldorf, Germany) was used for this purpose. Although a minimum of 12 participants per test was required, efforts were made to increase this number to improve statistical power. Finally, the minimum requirement was reached in all tests.

### 2.2. Participants

A total of 35 male amateur trail runners from a convenience sample with regular experience on uneven terrain participated in the study. Of the initial 40, five were excluded for various reasons (scheduling conflicts, illness, or injury). Recruitment was conducted in clubs located in Zafra, Los Santos de Maimona, and Cáceres through an online form. Each participant completed two test–retest sessions separated by 7 to 14 days.

Inclusion criteria required participants to be between 18 and 65 years of age, to practice trail running regularly at least twice per week, to be free of recent severe injuries anywhere on the body in the last 6 months. and to have the ability to understand and perform the tests. Individuals with neuromuscular disorders, recent severe injuries, or who used orthopedic aids were excluded.

On average, participants reported training 2–3 sessions per week on uneven terrain. According to the participant classification framework proposed by McKay et al. (2021) [29], this situates our sample within Tier 1 (Recreationally Active) and Tier 2 (Trained/Developmental), as runners trained consistently and some competed in local-level races. This characterization highlights that our findings are representative of amateur trail runners but cannot be generalized to elite or professional populations.

All participants signed informed consent approved by the Bioethics Committee of the University of Extremadura (code: 180//2025).

### 2.3. Materials and Instruments

Personal and anthropometric data of the participants (including height, weight, BMI, fat and lean mass, body circumferences, etc.) were collected using an Omron BF511 bioimpedance analyzer, an ADE MZ10042 stadiometer, and a measuring tape.

For the tests, the following instruments were used: a measuring tape with a precision of 0.1 cm, a One Plus Nord 3 5G smartphone with MediaTek Dimensity 9000 Octa-Core processor, 3.05 GHz, RAM 16 GB, ROM 256 GB, OxygenOS 13 (OnePlus. Shenzhen, China) (as goniometer the AngleMeter app (https://play.google.com/store/apps/details?id=com.stfactory.anglemeter; accessed on 16 February 2025) and stopwatch function), a YBT testing structure, a contact platform valid and reliable [30] (model A2, 420 × 590 mm, ChronoJump Boscosystem, Barcelona, Spain) factory-calibrated with a sampling frequency of 1000Hz, which estimates jump height based on the measurement of flight time, following the manufacturer’s instructions, and a MacBook Pro (Version OS X 10.14, 8GB, 2.30 GHz, Intel Core i5) with the Chronojump-BoscoSystem free software.

The following tests were administered:

- Lunge Test (Weight-Bearing Lunge Test, WBLT): The WBLT evaluates ankle dorsiflexion in a closed kinetic chain. It consists of touching the wall with the knee while placing the foot as far from the wall as possible without lifting the heel from the ground. Reduced ankle dorsiflexion is associated with a greater risk of lower-limb injuries during sports practice [31]. This is considered a reliable tool for assessing ankle dorsiflexion in healthy populations [32].
- Y Balance Test (YBT): The YBT is a tool designed to assess dynamic balance, core stability, postural control, and functional symmetry between lower limbs. It has proven to be highly reliable in healthy adults [33], military populations [34], and runners with intellectual disabilities [35], as well as a predictor of injury risk. The test consists of reaching as far as possible with the tip of the foot in three directions (anterior, posteromedial, and posterolateral) while maintaining single-leg balance without losing stability.
- Hop Test Battery: This battery includes 4 single-leg hop tests (Single Hop Test, Triple Hop Test, Crossover Hop Test, and 6m Timed Hop Test), which are used to evaluate explosive strength, dynamic stability, and inter-limb symmetry through the Limb Symmetry Index (LSI), particularly after musculoskeletal injuries [36,37], in order to determine the functional capacity of the injured leg compared to the healthy one. These tests have also been proven reliable in healthy populations [38,39,40].
- Countermovement Jump (CMJ): The CMJ is one of the most valid and reliable tests to assess lower-limb power across diverse populations, including elite athletes, adolescents, and runners from different athletic disciplines [41]. It shows significant correlations with markers of fatigue and with higher athletic performance among middle and long-distance runners [42]. The test consists of starting from a fully upright position, performing a rapid downward movement (countermovement), and then vertically jumping, taking advantage of the stretch-shortening cycle [43].

### 2.4. Test–Retest Procedure

Before the first session, all participants were informed of the study objectives. After signing the informed consent, anthropometric measurements and sociodemographic data were collected. Each participant performed a familiarization trial prior to the execution of each test. Although it was not possible, for logistical reasons, to measure all participants at the same time of day or to ensure fasting in every case, participants were strongly instructed to avoid strenuous physical activity and excessive fluid intake in the 12 h prior to assessment.

The two test–retest sessions were scheduled 7 to 14 days apart to avoid fatigue and learning effects. The execution order was fixed and not randomized: WBLT, YBLT, Single Hop, Triple Hop, Crossover Hop, 6m Timed Hop, and CMJ. This was carried out to minimize the influence of fatigue that might occur if more demanding tests were interspersed with less demanding ones. One research staff was responsible for administering the sociodemographic questionnaire, anthropometric and body composition measurements, and the WBLT. A second research staff conducted the remaining functional tests. Both followed standardized protocols, and each repeated the same tests in both sessions (test–retest), thereby ensuring methodological consistency.

### 2.5. Statistical Analysis

Once all results were obtained, the data were analyzed using SPSS (version 20.0) and Excel and were presented as means and standard deviations. Normality was assessed using Shapiro–Wilk which is recommended as the most appropriate method for small to moderate sample sizes (*n* < 50) (*p* ≤ 0.05).

Test–retest reliability was estimated using the ICC with a two-way mixed-effects model (3,1: two-way mixed, consistency, single rater) and its 95% confidence intervals. Munro’s classification was used to interpret the ICC: values between 0.50 and 0.69 as “moderate,” between 0.70 and 0.89 as “good,” and above 0.90 as “excellent” [44].

Absolute reliability was calculated using the Standard Error of Measurement (SEM) and the Minimum Detectable Change (MDC) with the following formulas:

SEM = SD × √1 − ICC

MDC = 1.96 × SEM × √2

Additionally, SEM% and MDC% were calculated according to Weir [27]. In line with previous literature, values below 10% for SEM and below 30% for MDC were considered acceptable thresholds for interpretation [45,46]. Bland–Altman plots [47] were also generated to assess agreement between the two measurements, using RStudio (Version 2024.12.1+563).

## 3. Results

Table 1 summarizes the main characteristics of the sample (*n* = 35), including the means and standard deviations of the anthropometric and physiological measurements obtained from all participants.

To ensure the reliability of the instruments and tests employed in this study, a test–retest reliability analysis was performed on the total sample (Table 2).

Table 2 reports the ICC values with their 95% confidence intervals, as well as SEM and MDC for each variable. The results indicated “good” to “excellent” reliability (ICC > 0.80) for the WBLT, the various Hop Tests, and the CMJ. In contrast, the YBT demonstrated lower reliability, with ICC values ranging from 0.554 to 0.732 and comparatively wider confidence intervals, indicating greater measurement variability.

**Figure 1:**
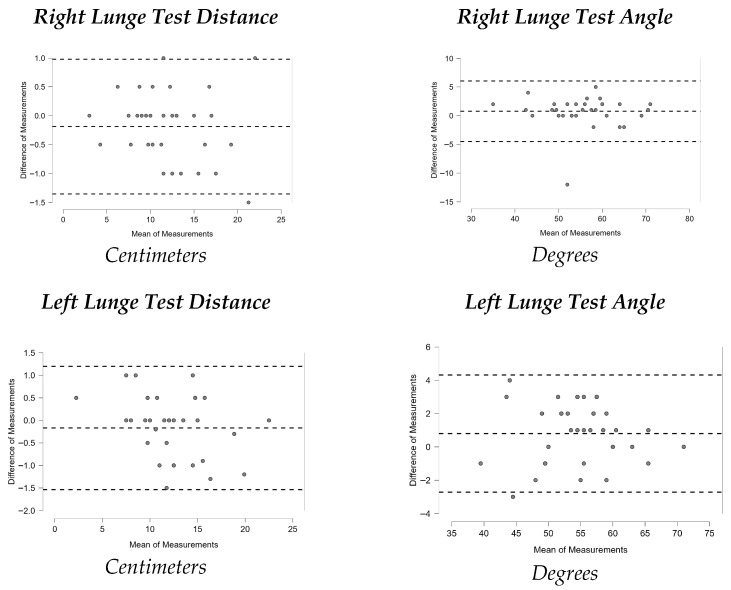
Bland–Altman plots Lunge Test (WBLT).

**Figure 2:**
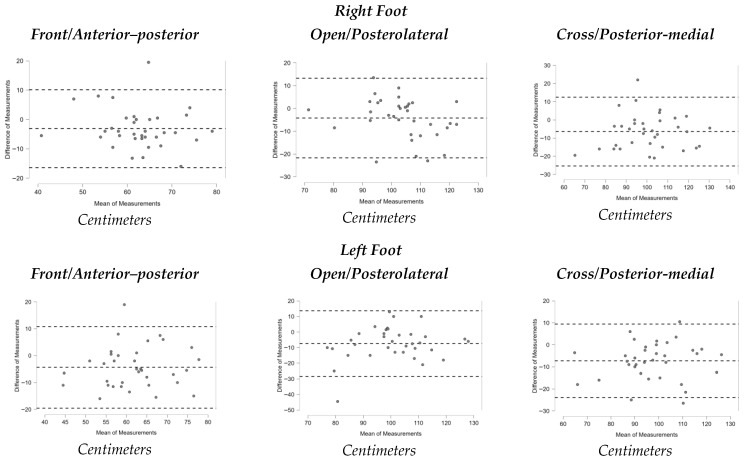
Bland–Altman plots Y Balance Test (YBT).

**Figure 3:**
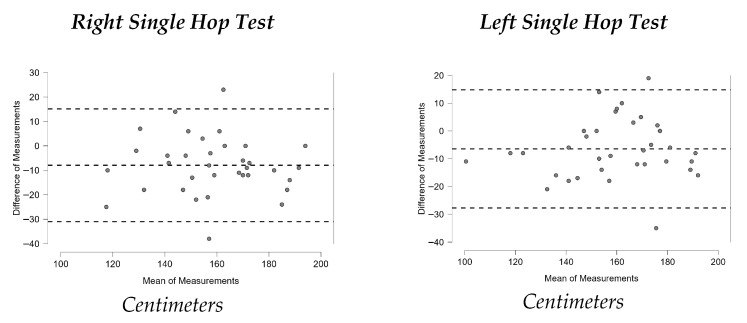
Bland–Altman plots Single Hop Test.

**Figure 4:**
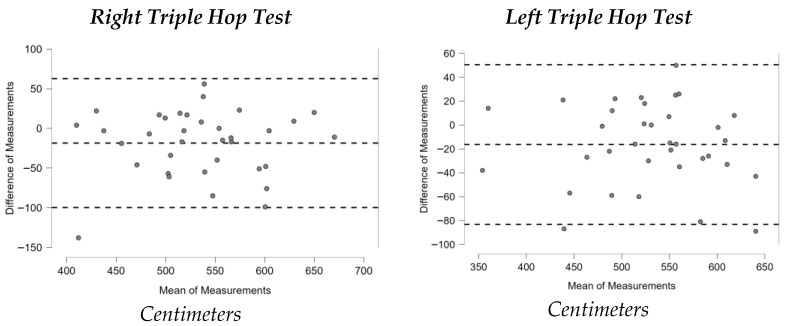
Bland–Altman plots Triple Hop Test.

**Figure 5:**
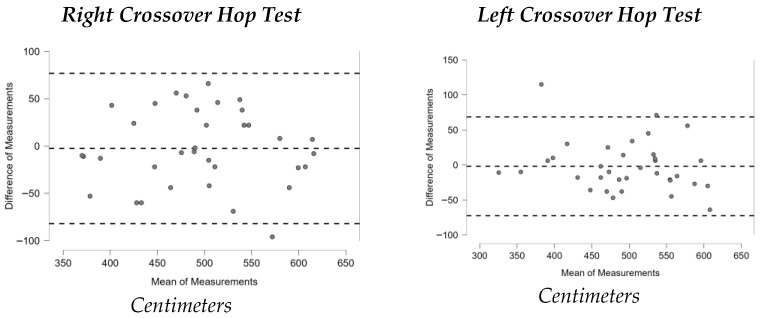
Bland–Altman plots Crossover Hop Test.

**Figure 6:**
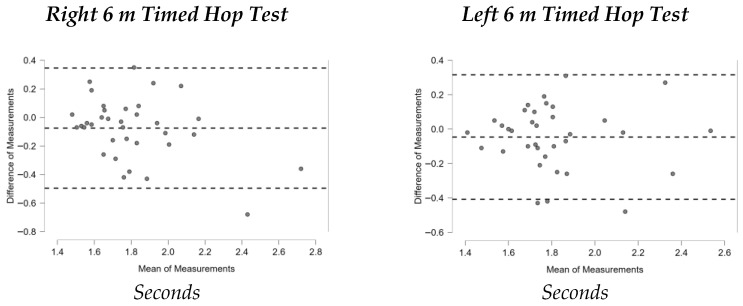
Bland–Altman plots 6m Timed Hop Test.

**Figure 7:**
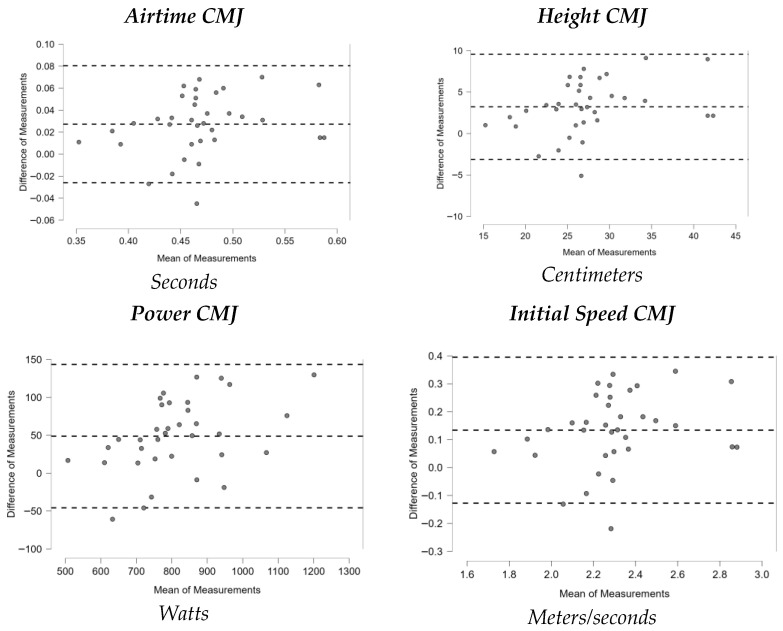
Bland–Altman plots CMJ.

Figure 1, Figure 2, Figure 3, Figure 4, Figure 5, Figure 6 and Figure 7 display the Bland–Altman plots for the tests analyzed.

Overall, the analysis of these plots revealed strong agreement between test and retest for most variables, especially in the ankle dorsiflexion test (Lunge Test) and the jump tests (Single Hop Test, Triple Hop Test, Crossover Hop Test, and CMJ), where the majority of data points fell within the 95% Limits Of Agreement (LOA). By contrast, the YBT and the 6m Timed Hop Test exhibited greater variability, with a higher proportion of data points falling outside the LOA.

## 4. Discussion

The purpose of the present study was to analyze the absolute and relative test–retest reliability of the Lunge Test, Y Balance Test, Single Hop Test, Triple Hop Test, Crossover Hop Test, 6m Timed Hop Test, and the Countermovement Jump in 35 amateur trail runners.

The main finding was that the majority of the tests demonstrated high inter-session reliability. These results support the use of these tests as robust methods for assessing functional performance in key domains such as ankle dorsiflexion, postural stability, dynamic balance, and lower-limb explosive strength in trail runners, even in contexts requiring high precision, such as rehabilitation programs or performance monitoring. Below, the findings for each test are discussed in detail.

The Lunge Test (WBLT) showed very high reliability, with ICC values of 0.990 for right-leg distance and 0.983 for left-leg distance, as well as 0.941 and 0.958 for right- and left-leg angular measures, respectively. Our ICCs for distance measures (0.983–0.990) are within the values previously reported in healthy individuals (0.97–0.99) [48], and people with ankle dysfunctions (0.93–0.99) [49], while the angular measures (0.941–0.958) are slightly lower than the highest values observed in the literature in healthy individuals (0.97–0.99) but still indicate excellent reliability. These findings therefore confirm the WBLT as a highly reliable tool for assessing functional ankle dorsiflexion in trail runners.

In contrast, the Y Balance Test (YBT) demonstrated only moderate reliability, with ICC values ranging from 0.554 to 0.732, SEM values between 6.52% and 9.79%, and MDC values between 18.06% and 27.14%. These relatively high error indices limit its sensitivity to detect change. This contrasts with other research reporting highly reliable ICC in military populations [34], healthy adults [33], and runners with intellectual disabilities [35]. One possible explanation is that the participants may have had limited familiarity with the YBT task, which requires practice to perform consistently; a recent review [50] indicate that 4–6 familiarization trials are typically needed to obtain reliable results and mitigate learning effects. In addition, trail running involves terrain-specific balance adaptations (uneven and unpredictable surfaces) that may not fully transfer to the standardized, flat-surface conditions of the YBT, potentially affecting performance stability in this population [51]. Finally, protocol- and measurement-related factors are known to influence YBT outcomes—such as the score calculation method, restrictions on arm movement, and the use of a dedicated YBT kit to standardize reach recording—[50]. In our study, although the test was performed barefoot and the average of three trials in each direction was used, the absence of restrictions on arm movement may have contributed to greater variability and increased measurement error. Therefore, in our sample, the YBT cannot be confirmed as a reliable method for assessing dynamic balance in amateur trail runners. The Single Hop Test demonstrated “good” reliability in both the right (ICC = 0.776) and left leg (ICC = 0.842). These results confirm its consistency for assessing lower-limb power and functional symmetry. While lower than values reported in some studies with healthy participants (ICC = 0.96) [38], our findings align more closely with research stratified by sex, where ICC ranged between 0.76 and 0.92 [39]. This suggests partial agreement with the literature, supporting the Single Hop Test as a reliable and objective measure in amateur trail runners.

Similarly, the Triple Hop Test demonstrated “good” reliability, with ICC values between 0.780 and 0.863. Although lower than those reported in studies with healthy subjects (ICC = 0.95) [38], our results align with findings from populations with musculoskeletal injuries (ICC = 0.84) [36]. Thus, the present data are broadly consistent with previous research.

Recent evidence suggests that hopping shares some biomechanical features with running, supporting its use as a functional test; however, the transfer between tasks is not perfect, and differences in motor strategy may influence performance consistency [52]. This partial overlap could explain why trail runners, whose training is more oriented toward irregular terrain rather than standardized horizontal hopping tasks, showed greater variability and consequently lower ICC values compared to other healthy populations or in sports such as basketball, where jumping-related movements are more specific and closely aligned with the demands of hop tests [53]. Moreover, test–retest variability in horizontal hop performance may have been amplified by limited familiarization and by allowing free arm movement. Although our protocol included one familiarization trial, it is possible that at least two would have been necessary to ensure greater consistency [54]. Similarly, because arm use was not standardized during the hops, variability between sessions may have increased, leading to lower ICC values [55].

The Crossover Hop Test also showed high reliability, with ICC values of 0.851 (right leg) and 0.884 (left leg). Prior studies have reported ICC values of 0.96 [38] and between 0.84 and 0.91 [39]. Our findings are therefore in agreement with the literature and confirm the Crossover Hop Test as a valid and reliable tool to assess lower-limb functional status, not only in trail runners but also in rehabilitation and return-to-sport settings.

The 6m Timed Hop Test yielded lower ICC values (0.691 for the right leg and 0.741 for the left leg), corresponding to “good” reliability for the right leg and “moderate” reliability for the left [44]. The greater variability may be attributed to manual timing with a smartphone device, which is subject to evaluator influence. Nevertheless, these values exceed those reported by Bolgla and Keskula [38] (ICC = 0.66), although more recent studies have shown higher reliability (0.82–0.92) [39]. Therefore, while reliable, this test requires stricter protocol control and more objective timing methods to reduce measurement error. In this sense, it is recommending the specific use of photocell systems, which have been demonstrated to reduce measurement error and bias, and are widely considered the gold standard for timing in sport performance, particularly for monitoring horizontal displacement [56].

Finally, the Countermovement Jump (CMJ) measured with a contact platform showed good reliability, with ICC values close to 0.75 across all parameters, the highest being Power (ICC = 0.894). The SEM averaged 5.84% and the MDC 16.18%, which are better than values reported in regional athletes [57] and basketball players [58]. Studies using validated instruments such as force plates or contact platforms, as in the present study, have also reported high reliability (ICC = 0.89–0.97) [58]. On the other hand, airtime (ICC = 0.755, SEM% = 5.79, MDC% = 16.06) and initial speed (ICC = 0.754, SEM% = 5.61, MDC% = 15.54) also showed good reliability, fully meeting the commonly accepted thresholds of SEM% < 10 and MDC% < 30. In contrast, although height was likewise categorized as having good reliability based on its ICC (0.753), it exceeded these thresholds (SEM% = 11.34, MDC% = 31.44) and therefore represents the least reliable CMJ-derived variable. These findings confirm the CMJ as a solid and reliable tool for assessing lower-limb explosive strength in amateur trail runners, provided validated and consistent measurement methods are used.

This study has several limitations. First, only inter-session reliability was assessed; thus, the reported reliability values may differ if measurements are performed within the same session. Second, in some tests—particularly jump tests—slightly lower scores were observed in the retest compared to the initial test. This may be related to factors such as fatigue in participants who arrived after running or reduced novelty and motivation during the second session. Third, no detailed data were collected on participants’ activity or training volume, which could have influenced performance outcomes. Fourth, this study only included male participants. This was ensured to reduce variability due to hormonal fluctuations associated with the menstrual cycle, which have been shown to influence strength, muscle function, and performance reliability in women [59,60]. As such, our results may not fully generalize to female populations. Fifth, fasting could not be strictly controlled. This may have introduced additional variability related to hydration status in bioimpedance data, although participants were instructed to avoid strenuous exercise and excessive fluid intake during the 12 h prior to the test. Sixth, the order of the tests was not randomized. A fixed sequence was deliberately chosen to minimize fatigue and, importantly, to ensure identical testing conditions across both sessions, which is critical in test–retest reliability designs. However, we acknowledge that this approach may introduce potential order effects. Seventh, the test–retest interval varied between 7 and 14 days. Although this was mainly due to scheduling issues by participants, it may have introduced additional variability, even though no substantial changes in performance were expected within this period. Eighth, the dorsiflexion angle and timing of the 6 m Timed Hop Test were assessed using a smartphone application and stopwatch, function, respectively. As these tools have not been validated for these specific measurements, the possibility of measurement error cannot be ruled out. Future studies should incorporate validated apps to minimize evaluator influence and improve measurement accuracy.

Future research directions include examining the reliability of these tests in elite trail runners, exploring potential sex-related differences in reliability and performance, validating these tests under real mountain conditions, correlating test outcomes with race performance, investigating their predictive value for injury risk, and evaluating intra-session and inter-rater reliability.

## 5. Conclusions

The relative reliability results obtained in this study demonstrate that most of the functional tests analyzed exhibit good-to-excellent reliability in a sample of amateur trail runners. In particular, the WBLT, Single Hop Test, Triple Hop Test, Crossover Hop Test, and Countermovement Jump showed high ICC values and acceptable SEM and MDC values, confirming their suitability for assessing ankle dorsiflexion, mobility, and lower-limb power. By contrast, the Y Balance Test and the 6 m Timed Hop Test demonstrated comparatively lower reliability and higher error indices. Therefore, these latter tests should be interpreted with caution and may require further refinement and protocol standardization before they can be confidently applied in amateur trail runners.

In summary, this study confirms that, provided standardized protocols and validated measurement tools are employed, these functional tests can be reliably used in amateur trail running. Their application allows not only the assessment of performance but also the detection of functional asymmetries and the prevention of musculoskeletal injuries associated with this sport. Accordingly, the present work provides a solid basis for integrating these tests into the monitoring and optimization of performance in amateur trail runners. Future research should extend these findings by including female and elite runners and by evaluating test reliability under real trail conditions, which may impose additional biomechanical and physiological demands.

## Figures and Tables

**Table 1:** Characteristics of amateur trail runners included in the study.

Variables	(*n* = 35)
Age (years)	40.49 (8.30)
Height (m)	1.73 (0.05)
Weight (Kg) *	71.93 (7.56)
Body Mass Index (kg/m^2^) *	24.12 (2.11)
Fat Mass (%) *	18.4 (4.34)
Lean Mass (%) *	39.29 (3.03)
Basal Metabolic Rate (Kcal) *	1638.26 (104.42)
Visceral Fat *	7.14 (2.41)
Waist Circumference cm)	81.34 (6.23)
Hip Circumference (cm)	93.64 (5)
Waist-to-hip Ratio	0.87 (0.44)
Right Arm Circumference (cm)	29.73 (2.74)
Left Arm Circumference (cm)	29.34 (2.78)
Right Thigh Circumference (cm)	55.68 (3.01)
Left Thigh Circumference (cm)	55.27 (3.38)
Right Calf Circumference (cm)	37.14 (1.81)
Left Calf Circumference (cm)	36.98 (1.77)

* Values obtained with the bioimpedancemeter Omron BF511.

**Table 2:** Absolute and Relative Reliability of the different variables.

Variable	Mean (SD Test) n = 35	Mean (SD Test) n = 35	*p*	ICC (95% CI)	SEM	SEM (%)	MDC	MDC (%)
Right Lunge Test Distance (cm)	11.90 (4.29)	12.09 (4.39)	0.074	0.990 (0.980–0.995)	0.434	3.62	1.2	10.03
Right Lunge Test Angle (°)	56.00 (7.97)	55.23 (8.03)	0.099	0.941 (0.886–0.969)	1.94	3.49	5.39	9.68
Left Lunge Test Distance (cm)	12.27 (3.72)	12.43 (3.94)	0.162	0.983 (0.967–0.991)	0.5	4.04	1.38	11.21
Left Lunge Test Angle (°)	54.89 (6.71)	54.09 (6.68)	0.013	0.958 (0.919–0.979)	1.37	2.52	3.8	6.98
Y Balance Test—Right Foot								
Front/Anterior–posterior (cm)	61.09 (7.95)	64.21 (8.83)	0.010	0.626 (0.377–0.791)	5.13	8.19	14.22	22.7
Open/Posterolateral (cm)	101.61 (10.72)	105.82 (13.29)	0.009	0.683 (0.459–0.826)	6.76	6.52	18.74	18.06
Cross/Posterior-medial (cm)	97.39 (15.42)	103.81 (14.86)	<0.001	0.724 (0.521–0.850)	7.95	7.91	22.05	21.92
Y Balance Test—Left Foot								
Front/Anterior–posterior (cm)	59.70 (9.34)	64.06 (8.81)	0.002	0.554 (0.278–0.746)	6.06	9.79	16.9	27.15
Open/Posterolateral (cm)	96.91 (14.89)	104.24 (12.71)	<0.001	0.595 (0.333–0.772)	8.78	8.73	24.34	24.2
Cross/Posterior-medial (cm)	94.19 (15.13)	101.45 (14.35)	<0.001	0.732 (0.533–0.855)	7.63	7.8	21.15	21.62
Right Single Hop Test (cm)	154.63 (20.41)	162.57 (20.92)	<0.001	0.776 (0.603–0.880)	9.78	6.17	27.11	17.09
Left Single Hop Test (cm)	156.23 (22.48)	162.71 (21.66)	0.001	0.842 (0.711–0.917)	8.77	5.5	24.32	15.25
Right Triple Hop Test (cm)	524.94 (67.92)	543.49 (66.24)	0.012	0.780 (0.608–0.882)	31.46	5.89	87.21	16.33
Left Triple Hop Test (cm)	520.94 (70.04)	537.29 (72.21)	0.008	0.863 (0.748–0.928)	26.33	4.98	72.97	13.79
Right Crossover Hop Test (cm)	494.97 (73.29)	497.54 (73.75)	0.710	0.851 (0.727–0.922)	28.38	5.72	78.66	15.85
Left Crossover Hop Test (cm)	494.89 (70.09)	496.80 (77.05)	0.755	0.884 (0.783–0.939)	25.06	5.05	69.45	14.01
Right Timed 6m Hop Test (seg)	1.77 (0.24)	1.84 (0.32)	0.051	0.691 (0.471–0.831)	0.16	8.62	0.43	23.9
Left Timed 6m Hop Test (seg)	1.79 (0.26)	1.83 (0.27)	0.263	0.741 (0.548–0.860)	0.13	7.45	0.37	20.65
Airtime CMJ (seg)	0.48 (0.06)	0.46 (0.05)	<0.001	0.755 (0.568–0.868)	0.03	5.79	0.08	16.06
Height CMJ (cm)	28.91 (6.77)	25.68 (5.69)	<0.001	0.753 (0.566–0.867)	3.1	11.34	8.58	31.44
Power CMJ (W)	837.60 (156.08)	788.81 (135.45)	<0.001	0.894 (0.802–0.945)	47.46	5.84	131.54	16.18
Initial Speed CMJ (m/s)	2.37 (0.28)	2.23 (0.24)	<0.001	0.754 (0.567–0.867)	0.13	5.61	0.36	15.54

SD: Standard Deviation; ICC: Intraclass Correlation Coefficient; CI: Confidence Interval; SEM: Standard Error of Measurement; MDC: Minimal Detectable Change.

## Data Availability

The data will not be shown publicly, as patients will give their consent for the information to be kept confidential.

## Abbreviations

The following abbreviations are used in this manuscript:

BMI	Body Mass Index
CI	Confidence Interval
CMJ	Countermovement Jump
ICC	Intraclass Correlation Coefficient
LOA	Limits Of Agreement
MAS	Maximal Aerobic Speed
MDC	Minimal Detectable Change
SD	Standard Deviation
SEM	Standard Error of Measurement
WBLT	Weight-Bearing Lunge Test
YBT	Y Balance Test
